# Atypical case of tuberous sclerosis with isolated neurologic findings: A case report

**DOI:** 10.1002/ccr3.9379

**Published:** 2024-09-03

**Authors:** Shritik Devkota, Om Prakash Bhatta, Arun Kalikote, Prakash Gyawali, Samiksha Lamichhane

**Affiliations:** ^1^ Department of Radiodiagnosis and Imaging Anil Baghi Hospital Punjab India; ^2^ Department of Radiodiagnosis and Imaging Postgraduate Institute of Medical Education and Research Chandigarh India; ^3^ Department of Internal Medicine Nova Hospital Dhangadhi Nepal; ^4^ Rani Primary Health Centre Biratnagar Nepal; ^5^ Department of Emergency Medicine Sukraraj Tropical and Infectious Hospital Kathmandu; ^6^ Department of Radiodiagnosis and Imaging BP Koirala Institute of Health Sciences Dharan Nepal

**Keywords:** genetic disorder, subependymal nodule, TSC1, TSC2, tuberous sclerosis complex

## Abstract

Tuberous sclerosis (TSC) is an autosomal dominant neurocutaneous disorder. This case highlights rare isolated neurologic finding in a TSC patient emphasizing the need for heightened suspicion even in the absence of any cutaneous findings and family history.

## 1 INTRODUCTION

Tuberous sclerosis complex (TSC) is a rare inherited neurocutaneous disorder with autosomal dominant inheritance. It is characterized by diverse features involving multiple organ systems, including the development of benign hamartomas in the brain, eyes, heart, lungs, liver, kidneys, and skin.^1^, ^2^ While Bourneville provided the first detailed description of the neurological symptoms and gross pathology in the central nervous system in 1880, it is important to note that this disorder is rare, and estimated to have incidence of 1 in 10,000 live births. TSC affects all age groups and races with no gender predilection.^3^, ^4^, ^5^

TSC was first depicted in 1835 when Pierre Francis Olive Rayer published color drawing showing small erythematous papules that resemble “facial angiofibroma.” In 1862, Von Recklinghausen then reported cases of cardiac myomatas and cerebral sclerosis in newborns who died just after birth. Bourneville then noted partial and generalized seizures, sclerotic areas on cerebral convolutions, vesiculo‐papular eruption on nose, renal tumors on a mentally subnormal 15 year old girl. He then coined the term “sclerose tubereuse des circonvolutions cerebrales.”^6^ These are some of the earliest evidences of manifestations of tuberous sclerosis complex, when it was not completely understood.

Mutations in the TSC1 or TSC2 gene are identified as the cause of TSC.^7^, ^8^ This discovery was made in the 1990s. These mutations dysregulate the mammalian target of rapamycin (mTOR) signaling cascade, leading to abnormal cell proliferation and the formation of hamartomas with multisystem involvement.^9^ With understanding of these disease pathology, mTORC1 inhibitors like everolimus, sirolimus has been approved for various manifestations of TSC.^10^ Recently, genetic testing to diagnose tuberous sclerosis complex has been introduced and with this, a new diagnostic criteria depending on genetic test has been accepted as well.

The diagnosis of TSC is made by the “TSC diagnostic criteria consensus statement,” which consists of “clinical diagnostic criteria” using clinical signs and radiologic findings, and “genetic diagnostic criteria” by detecting pathogenic TSC gene mutations.^11^

As a rare disorder, isolated neurological findings may be a rare presentation of TSC necessitating a high degree of suspicion. Here, we present a case of TSC with isolated neurological findings presenting with hypokalemia and seizures diagnosed by imaging findings.

## 2 CASE PRESENTATION

We present the case of an 18‐year‐old male who presented with fever, myalgia, and generalized weakness for 7 days. Upon investigation, a potassium level of 2.9 was noted, leading to admission for the correction of hypokalemia. No abnormalities were observed in other blood parameters (Table 1). However, on day 1 of admission, the patient suddenly experienced one episode of left‐sided complex partial seizure with postictal altered consciousness.

**TABLE 1:** Summary of laboratory investigation.

	Parameters	Results
Hematology	Random glucose	110 mg/dL (RR:70‐140 mg/dL)
	Total leukocyte count	8000 cells/mm^3^ (RR: 4000–11,000)
	Mean cell volume	88 femtoliters (RR: 80–100 femtolitres)
	Platelets	250,000 cells/mm^3^ (RR: 150000–450,000 cells/mm^3^)
	Uric acid	3 mg/dL (RR: 2.5‐7 mg/dL)
	Urea	18 mg/dL (RR: 5–20 mg/dL)
	Creatinine	0.7 mg/dL (RR:0.7–1.3 mg/dL)
	Sodium	138 meq/L (RR:135–145 meq/L)
	Potassium	**2.9 meq/L (RR: 3.5–5.5 meq/L)**
	Total bilirubin/direct bilirubin	0.8/0.1 mg/dL (RR: 0.1–1.2 mg/dL, <0.3 mg/dL)
	ALT/AST	25/22 (RR: 4–36/ 8–33)
	Creatine kinase	160 units/L (RR: 22–198 units/L)
Hormone Studies	Thyroid stimulating hormone	2.58 mIU/L (RR: 0.5–5 mIU/L)
	Free‐T4 (Thyroxine)	1.2 ng/dL (RR: 0.9–2.3 ng/dL)
	Vit D3 (25‐OH)	10.7 ng/mL (RR: 20–40 ng/mL)
	Ca	9.2 mg/dL (RR: 8.5–10.2 mg/dL)
Arterial blood gas	pH	7.38 (RR: 7.35–7.45)
	PCO_2_	40 mmHg (RR:35–45 mmHg)
	HCO_3_	24 meq/L (RR:22–28 meq/L)

Abbreviation: RR, Reference Range.

*Note:* Bold indicates hypokalemia.

## 3 DIAGNOSTIC WORK UP

**FIGURE 1:**
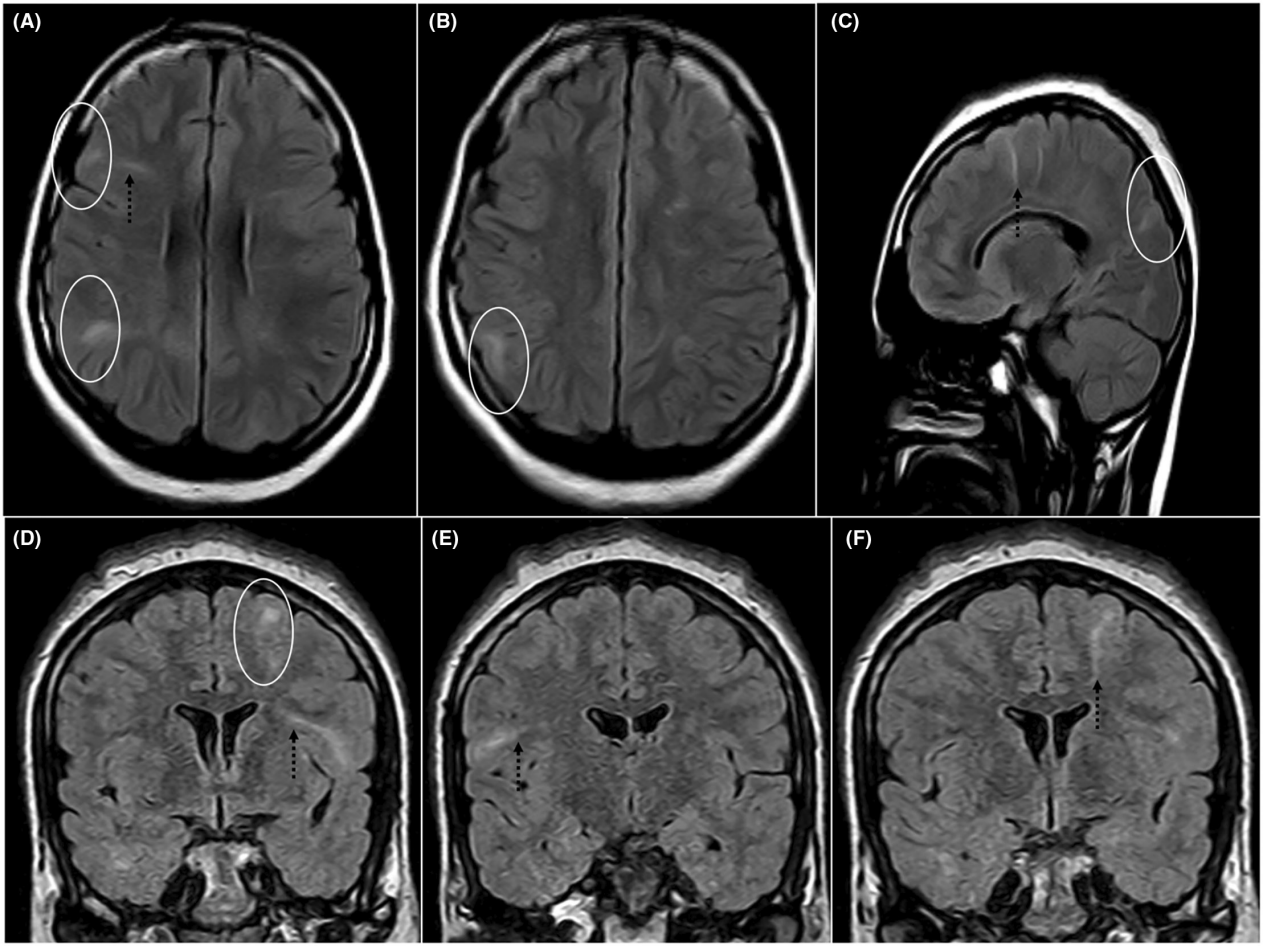
Axial (A, B), sagittal (C), and coronal (D, E, F) FLAIR images showing cortical and subcortical areas of FLAIR hyperintensities (white circles) suggesting tubers and linear hyperintensities extending from cortex suggesting radial bands.

**FIGURE 2:**
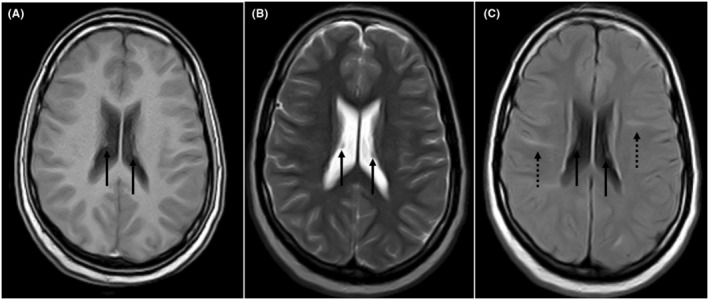
Axial T1W (A), T2W (B), and FLAIR (C) images showing small subependymal nodules (black arrows) and FLAIR hyperintense radial bands (dotted arrow, C).

**FIGURE 3:**
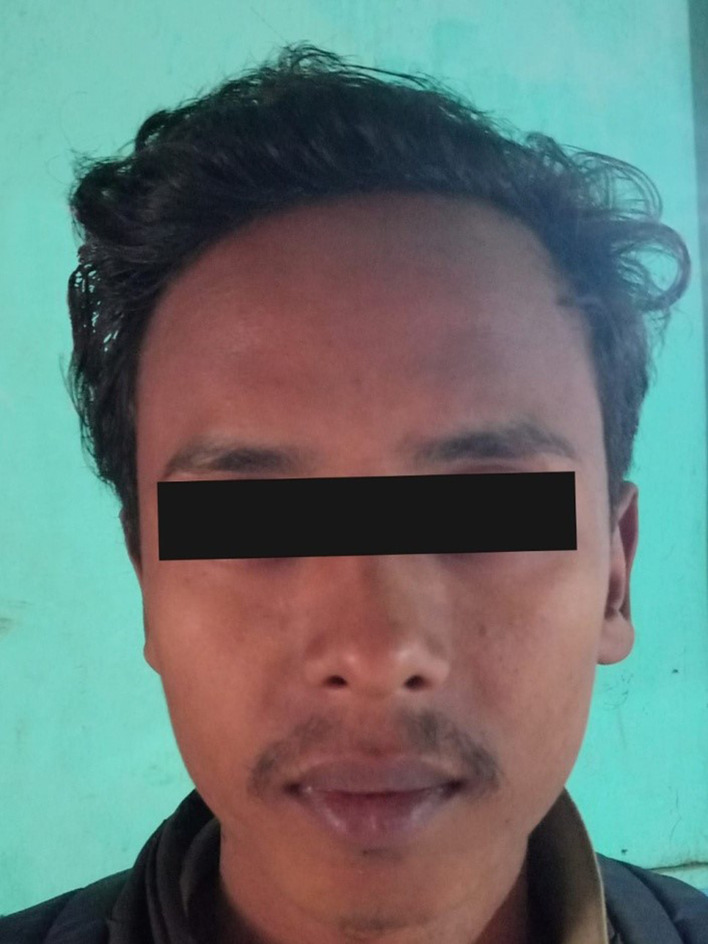
Absence of any facial features in the index case.

Following this new event, an MRI was performed, revealing radial bands, subependymal nodules, and cortical tubers. These findings fulfilled two major clinical criteria of tuberous sclerosis, allowing us to make a definitive diagnosis. Surprisingly, upon further examination, no cutaneous manifestations were noted, making this case intriguing. A detailed examination of the patient's family history did not identify any prior cases of tuberous sclerosis. Ultrasonography, echocardiography, and fundoscopy were performed, but no significant findings were observed. A CSF examination was done which was normal. The patient was started on tablet oxcarbazepine 300 mg twice a day and kept in regular follow‐up. The findings of the MRI are given in Figures 1 and 2. Absence of cutaneous features in face can be noted in Figure 3.

## 4 DIFFERENTIAL DIAGNOSIS

While cortical tubers, radial bands, and subependymal nodules are characteristic imaging findings in TSC, we performed a clinical and radiological differential diagnosis to rule out other potential causes. Multiple sclerosis (MS) was excluded due to the absence of pericallosal signal changes with the characteristic “Dawson finger” appearance and a normal spinal cord MRI. Similarly, neuromyelitis optica (NMO) was ruled out by the lack of optic nerve involvement on imaging and the absence of any related optic nerve symptoms in the patient. Finally, focal cortical dysplasia (FCD) can occasionally mimic isolated cortical and subcortical tubers with radial bands. However, the absence of cortical thickening and any abnormal gyral‐sulcal pattern on imaging argued against FCD.

## 5 OUTCOME AND FOLLOW UP

Our patient was started on tablet oxcarbazepine 300 mg twice a day and kept on regular follow up. Patient has recovered well and has not reported any seizures in 3 months.

## 6 DISCUSSION

Tuberous Sclerosis is an autosomal dominant neurocutaneous syndrome, with high penetrance but variable expressivity.^12^ Our case does not have a family history of tuberous sclerosis.

Cutaneous manifestation is noted in nearly all patients and in past literature, features like hypopigmented macules have been noted in up to 97% of cases of children with tuberous sclerosis.^13^ But the lack of cutaneous findings in this patient doesn't align with the usual pattern of tuberous sclerosis. This emphasizes importance of high degree of suspicion for TSC even without cutaneous features. Since affected individuals can present with various clinical patterns and it is a progressive disease, it reflects the importance of thorough evaluation and regular screening of relevant conditions, once diagnosis is made. D. P. E. Kingsley has reported 18 cases out of 110 cases presenting with radiographic features but no clinical features. Out of them, two patients went on to develop skin findings later on.^14^ This also highlights importance of continuing follow‐up in TSC.

This patient had a complex partial seizure, consistent with common clinical presentation in tuberous sclerosis. This adds to the existing literature on the high prevalence of seizure and epilepsy in patients with tuberous sclerosis. But majority of patient with tuberous sclerosis present with infantile spasms or seizure onset on early years of life. Our patient had no history of seizure before and has presented with first episode of seizure at 18 years of age. Nevertheless, it has been reported that majority of TSC patients with even just one episode of seizure develop epilepsy.^15^ This adds to the rationale behind anti‐seizure medication after seizure onset in tuberous sclerosis. On our case, we started patient on oxcarbazepine and kept him on regular follow up. Patient has recovered well and has not reported any seizures in 3 months.

### 6.1 LIMITATIONS

This case report acknowledges some limitations. Despite an extensive workup, only neuroimaging findings were present to support the diagnosis of tuberous sclerosis (TSC) at presentation. Genetic testing, which could have definitively confirmed the diagnosis, was not performed. Additionally, the patient's follow‐up was limited to 3 months, demonstrating improvement in seizure episodes. A more extensive follow‐up period might reveal the development of facial or other extra‐neurological manifestations of TSC at a later stage.

## 7 CONCLUSION

In conclusion, this case highlights the diverse and often atypical presentations of TSC. Our patient's isolated neurological symptoms, including hypokalemia and seizures, in the absence of the classic cutaneous manifestations or family history, underscore the importance of maintaining a high index of suspicion for TSC. Clinicians should consider TSC in the differential diagnosis of any unexplained neurological presentation. Prioritizing early recognition through a comprehensive assessment that incorporates both clinical and genetic criteria is crucial for timely management and improved patient outcomes. Due to the rarity of TSC and the potential for misdiagnosis, cases like this one are vital to increase awareness of the condition and ensure accurate diagnosis and optimal patient care.

## AUTHOR CONTRIBUTIONS

**Shritik Devkota:** Conceptualization; formal analysis; investigation; methodology; resources; supervision; validation; visualization. **Om Prakash Bhatta:** Data curation; formal analysis; investigation; software; supervision; writing – original draft. **Arun Kalikote:** Data curation; formal analysis; investigation; software; supervision; writing – original draft. **Prakash Gyawali:** Conceptualization; formal analysis; investigation; supervision; validation. **Samiksha Lamichhane:** Conceptualization; formal analysis; investigation; resources; supervision; validation.

## FUNDING INFORMATION

No sources of funding.

## CONFLICT OF INTEREST STATEMENT

Authors declare no conflict of interest.

## ETHICS STATEMENT

Patient anonymity is maintained and consent was obtained for publication from the patient.

## CONSENT

Written informed consent was obtained from the patient to publish this report in accordance with the journal's patient consent policy.

## Data Availability

Anonymized data is accessible upon request from the corresponding author.
